# Jejunal Diverticulitis: A Rare Cause of Left Lower Quadrant Pain

**DOI:** 10.5334/jbr-btr.859

**Published:** 2015-09-15

**Authors:** W. Van Dessel

**Affiliations:** 1 Department of Radiology, Universitaire Ziekenhuizen Leuven, Leuven, Belgium

A 48-year-old female patient presented to the emergency department with increasing abdominal pain in the left hemi-abdomen, nausea and fever for two days. Clinical examination revealed mild rebound tenderness in the left lower quadrant. Laboratory tests showed leukocytosis and a raised C-Reactive Protein level.

A CT-scan with oral, rectal and intravenous contrast was performed showing 2 jejunal diverticula (arrows in Fig. A & B) with important infiltration of the surrounding mesenteric fat (curved arrow in Fig. A & B) and wall thickening of the affected jejunal limb (arrow in Fig. C). The diagnosis of acute jejunal diverticulitis was made.

**Figures A–C:**
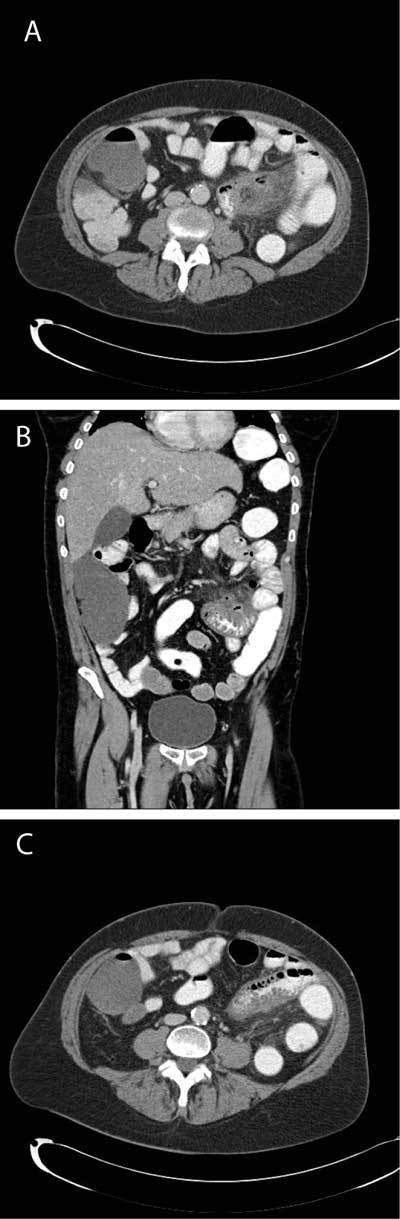


## Comment

Diverticula affecting the small bowel are very uncommon, especially when compared to colonic diverticula. The incidence is estimated to be less than 2%, mostly exclusively diagnosed in adults with predominance for the male population (2:1). Diverticula of the small bowel and colon, except for a Meckel diverticulum, are considered to be of the pulsed type, consisting of thin walled mucosal outpouchings through areas of weakness in the muscular layer. They are usually found along the mesenteric side of the affected small bowel.

Symptoms are often non-specific. Other more common pathologies are usually suspected clinically, such as appendicitis, colonic diverticulitis, cholecystitis, pancreatitis etc. Since failed diagnosis or delayed treatment can be associated with high morbidity, one should be familiar with the pathology and be able to exclude it.

Typical CT imaging appearance of jejunal diverticulitis shows a luminal outpouching containing gas or faeces-like material, together with associated signs of acute inflammation like bowel wall thickening, edematous stranding of the surrounding mesentery and thickening of the adjacent fascia. Signs of perforation can be hard to detect and should be looked for carefully.

Differential diagnosis of focal thickening and adjacent inflammation of the small bowel include intestinal malignancies, foreign body ingestion, focal Crohn’s disease, medication-induced ulceration and traumatic hematoma. Treatment of choice is surgical resection to prevent recurrence or other complications such as perforation. In some cases, treatment can be conservative.

## Competing Interests

The author declares that they have no competing interests.
